# Personal continuity and access in UK general practice: a qualitative study of general practitioners' and patients' perceptions of when and how they matter

**DOI:** 10.1186/1471-2296-7-11

**Published:** 2006-02-24

**Authors:** Bruce Guthrie, Sally Wyke

**Affiliations:** 1 Tayside Centre for General Practice, University of Dundee, MacKenzie Building, Kirsty Semple Way, Dundee DD2 4BF, UK; 2 Self Care Alliance, University of Stirling, Room 4T10, Department of Nursing and Midwifery, Stirling FK9 4LA, UK

## Abstract

**Background:**

Personal continuity is a core value for family practice, but policy and performance targets emphasise other aspects of care, particularly waiting times for consultation. This study examined patient and general practitioner (GP) perceptions of the value of personal continuity and rapid access, and the relationship between them.

**Methods:**

Qualitative analysis of semi-structured interviews with a purposive sample of 16 GPs and 32 patients in the Lothian region of Scotland, to identify whether, how, why and in which circumstances personal continuity and rapid access were valued.

**Results:**

From the patients' perspective, what mattered was 'access to appropriate care' depending on the problem to be dealt with. For a few patients, rapid access was the only priority. For most, rapid access was balanced against greater involvement in the consultation when seeing 'their' trusted doctor, which was particularly valued for chronic, complex and emotional problems. GPs focused on the value of personal continuity in the consultation for improving the diagnosis and management of the same kinds of problem. GPs did not perceive enabling access to be a core part of their work. There was little evidence that GPs routinely discussed with patients when or how personal continuity and access should be balanced.

**Conclusion:**

'Access to appropriate care' from the patients' perspective is not fully addressed by GPs' focus on personal continuity, nor by performance targets focused only on speed of access. GPs need to make enabling access as much a part of their core values as personal continuity, and access targets need to be based on less simplistic measures that account for the appropriateness of care as well as speed of access.

## Background

Personal continuity of care, or an ongoing therapeutic relationship between a patient and their clinician, has been a core value for general and family practice since the 1950s [1-5]. However, its relevance in the 21^st ^century has been questioned, particularly in countries where health service organisation makes it difficult to achieve [6,7]. Even in countries like the UK and the Netherlands where personal continuity has been stronger, health service reorganisation such as new out of hours arrangements, practices growing in size, and the development of multi-disciplinary primary care teams have tended to reduce the scope for personal continuity [8]. This reflects that there are other valued aspects of healthcare including quality of clinical process and outcome, and increasingly rapid and convenient access to care.

In the United Kingdom (UK), the general practice contract provides financial incentives for practices to achieve nationally set targets on speed of access and the same targets are used for performance management of Primary Care Trusts [9,10]. The focus on access reflects public concern about waiting times in primary care, and there has been widespread implementation of 'Advanced Access', an organisational intervention to reduce waiting times for appointments that originated in the United States (US) [11,10]. However, there is some evidence that in at least some of its implementations, Advanced Access reduces personal continuity for patients who want it [10,12]. Similarly, other organisational change in the US, including forced change in healthcare provider by insurance plan and reduced admission rights for office-based doctors, are seen as threatening personal continuity in general and family practice [2].

There is evidence to justify both GPs' emphasis on personal continuity and policy focus on access. Most patients value seeing a general or family practitioner that they know, particularly those who are older, sicker and consulting more frequently [13-15]. Research consistently shows an association between patient satisfaction and personal continuity [16,2,8,17], with some evidence of an effect on patient outcomes such as hospitalisation [17,2]. Equally though, long waiting times for appointments are a major source of patient dissatisfaction [18,19], and many patients prioritise access over personal continuity for urgent or more episodic problems [14,15,20].

Both family practitioner organisations and policymakers can therefore legitimately claim to be acting according to patients' wishes. However, it is less clear how access and personal continuity are balanced by GPs in everyday practice (as opposed to the views of those writing about 'core values' in the professional literature [1,3]) or by patients actually receiving care for particular problems (as opposed to answering surveys using hypothetical vignettes [14,15]). The aim of this paper is to examine whether, why and when general practitioners and their patients value personal continuity or rapid access to care, and to draw out the implications of these findings for the development of primary care services.

## Methods

The study used qualitative analysis of semi-structured interview data to investigate the place of personal continuity in relation to other valued aspects of care including access. Data collection took place in Lothian, Scotland in 1999–2001.

The initial phase of the study used convenience sampling to identify and interview 6 GPs and 4 patients, with the main phase of the study then using purposive sampling to ensure heterogeneity of participants. Ten practices were sampled, to include both larger and smaller practices, and those serving populations with a range of socioeconomic deprivation. One GP was randomly sampled from each practice and asked to participate. Because patients with and without chronic illness were expected to have different views, the participating GP was asked to recruit patients from three groups – those with no chronic disease, those with hypertension and those with diabetes. To avoid only recruiting patients that GPs had close relationships with, GPs were asked to recruit at least one person with chronic disease that they 'knew well', and one person they 'had some knowledge of, but wouldn't say they knew well'. Details of all participants and those declining to take part are given in tables 1, 2, 3.

**Table 1:** Characteristics of practices for participants and non-participants

	No of GPs participating (n = 16)	No of GPs declining to take part (n = 7)	No of patients participating (n = 32)	No of patients declining to take part (n = 11)
**Practice listsize**				
<2500	2	3	5	4
2500–4999	2	1	6	0
5000–7499	1	0	4	0
7500–9999	8	1	14	7
≥10 000	3	2	3	0
				
Practice deprivation*				
< -1.72	5	1	9	0
-1.72 to -0.35	3	0	5	1
-0.34 to 0.61	4	3	9	3
>0.61	4	3	9	5
				
**Practice training status**				
Training	8	3	12	7
Non training	8	4	20	4

* Carstairs score (quartiles within Lothian), Negative scores are more affluent, positive scores more deprived

**Table 2:** Individual characteristics of general practitioner participants and non-participants

	No of GPs participating (n = 16)	No of GPs declining to take part (n = 7)
**Age**	10	Not known
20–44	5	
45–64	1	
65 and over		
		
**Sex**		
Female	7	Not known
Male	9	
		
**GP employment**		
Full time	13	Not known
Part time	3	

**Table 3:** Individual characteristics of patient participants and non-participants

	No of patients participating (n = 32)	No of patients declining to take part (n = 11)
**Age**	9	Not known
20–44	11	
45–64	12	
65 and over		
		
**Sex**		
Female	17	5
Male	15	6
		
**Patient's current preference for seeing particular GPs**^+^		
Currently preferred to see the interviewed GP	16	Not known
Currently preferred to see another GP	5	
Didn't have a preferred GP at time of study	7	
		
**Patient's estimate of no of consultations in the last year**^+^		
0	1	Not known
1–4	12	
5–9	12	
10 or more	3	

+ Only for patients in main phase of study

Initial contact was made by telephone for GPs, and in writing with telephone follow up for patients, and all those approached received a Participant Information Sheet which included information about the study, that participation was entirely voluntary, that withdrawal at any stage was possible, and contact details for an independent advisor. Before each interview, any questions about the study were discussed in full and a consent form approved by the ethics committee signed. At the end of the interview, the participant's willingness for the data collected to be used in the study was verbally confirmed and any further questions about the study discussed.

Interviews were conducted by BG at a convenient location for participants (usually the patient's home or the general practitioner's surgery) in 1999–2001. Most lasted between 45 and 75 minutes, and all bar one were audio-taped, transcribed and anonymised. Initial topic guides were developed based on the literature, modified during the initial phase, and evolved throughout the study according to early analysis. Examples of those used at the start of the main phase of the study are shown in additional file 1, the main later modification being further probes about 'trust' and 'confidence' if participants did not spontaneously mention them [see Additional file 1]. Topic guides were used as a prompt to the interviewer to ensure that relevant areas were covered, rather than being a list of questions to be asked in order and were used flexibly depending on what the participant themselves identified as important rather than solely being focused on continuity. Since we wanted to ensure that doctors' interviews were focused on care for particular individuals, patients were interviewed first, and their permission sought for their care to be discussed in the GP interview. All but one patient agreed to this. All data, including the initial phase interviews, were analysed.

Data management and analysis was facilitated using NVivo software as an indexing and coding tool [17]. Validity was ensured by repeated reading of whole transcripts to keep the analysis comprehensive; by the use of a form of constant comparison using an active search for counter examples to emerging analysis, and by modification of the topic guide in response to early analysis. Reliability was ensured through regular meetings between the main analyst (BG) and two other researchers to discuss all analytical notes written, shared analysis of a sample of transcripts, and disagreements being resolved by discussion and re-analysis [21-23]. No new themes emerged during analysis of interviews with GPs and patients in the final two practices, at which point it was considered that saturation had been achieved.

This paper presents the results of an interpretative, thematic analysis of how participants discussed access and personal continuity, and their relationship to each other. All respondent names have been changed, and other strong identifiers altered in the quotes used.

## Results

The main phase of the study successfully recruited patients according to the sampling frame, and included patient-GP pairs with no longitudinal relationship and little knowledge of each other, as well as those with long-term close relationships (tables 1, 2, 3).

### Personal continuity and access from the patients' perspective

Discussion of personal continuity and access was intertwined in the patient interviews. Patients talked at some length about how they accessed general practice, a process usually requiring a negotiation with a receptionist to make an appointment, although sometimes by turning up and waiting in an open surgery. The ability to be seen quickly was valued by all patients, and its necessity for problems perceived to be urgent was taken for granted. However, most patients balanced 'when to be seen' against 'who to see', depending on the problem to be discussed. Four patients said they had no preference for which GP they saw under any circumstances (two patients without chronic disease, and one each with hypertension and diabetes). For the rest, consultation for chronic or emotional problems were most commonly mentioned as making 'who to see' more important, and most of those with chronic problems said they tried to see 'their' GP, unless this was overridden by a problem perceived to be urgent. Although most patients without an ongoing chronic problem said they currently prioritised rapid access over personal continuity, all but two said that there had been circumstances in the past when they had prioritised personal continuity (for example, for antenatal care, or follow-up of a now resolved problem), and could see circumstances where they would do so again (table 4).

**Table 4:** Patients' discussion of access and personal continuity intertwined, with 28 of 32 patients balancing each against the other depending on the problem to be dealt with

'I have always been impressed by that particular GP, Dr Comrie. He listens without wasting a lot of time. And the impression I get is that he doesn't treat me as another number, he will talk the position over. ... I normally see him. Always. *BG And perhaps, thinking if Dr Comrie wasn't available*? Well I would see someone else. If it was serious enough.' **Mr C2 (high blood pressure)**

'I'm quite happy to see any doctor ... if it's a general thing that I thought, 'I'm not feeling that great, I've got the cold or something'. If it was something that was worrying me or I wasn't sure about, I would possibly go back to the doctor that I seen during my pregnancy ... because I felt I really trusted him.' **Mrs T1 (no chronic disease)**

'It's necessary to have a good personal relationship and I think that's quite an important feature, to me anyway, might not be to everybody, but it's something I look for. … There's a deeper level that you feel there is an understanding between you, at a subliminal almost level, it's not just conversation, it's not just professional etiquette, it's like, the guy relates to me, I understand what he's talking about, I believe he understands what I'm saying, and we get on. … [but] If I have to be seen quickly, I'll put that to one side obviously. You can't just say "Well I demand to see Dr X", I mean, we're thinking about the Dr Findlay days when the Doctor would grab his black bag and rush out to Mrs So-and-so, because she was having a fit of the vapours or something, oh no, no, forget that. No, you would put that to one side and say if it's something serious, 'What I need is a qualified medical practitioner to have a look at this right now, I don't care who it is.' **Mr H2 (high blood pressure)**

'I normally work and I find that trying to get an appointment can be real pain in the neck unless there is an open clinic, which is why it would be far better for me, if I could go along in an evening, and that's not something that's available ...
*BG Perhaps just thinking that through, one way that you could provide evening care would be to be seen in the evenings by doctors working on shifts, what you would lose from that would be seeing your own doctor, would be that something that was important to you?* Not particularly.' **Mrs H1 (no chronic disease)**

Decisions about 'who to see' were largely driven by the value placed on personal continuity or an ongoing relationship with a particular GP. Less commonly, patients said they tried to avoid particular GPs because of unsatisfactory consultations with them in the past. For those who valued it, personal continuity was said to make consultations feel more comfortable, made it easier to ask questions and be involved in the consultation and decision making. Greater ease was reported in the consultation because: patients did not have to pay as much attention to presenting themselves as legitimate users of services; they trusted 'their' GP to take responsibility for them over time as a whole person, rather than simply dealing with the immediate problem to hand; and finally consultations for chronic conditions were also said to be more efficient because patients didn't have to repeat their whole story each time (table 5). Patients rarely talked explicitly about personal continuity leading to better diagnosis or management of problems, although some perceived that personal continuity meant that their treatment was more tailored to their individual circumstances.

**Table 5:** Why does personal continuity matter from the patient's perspective?

'Well, there's a link comes and you've a got a confidence because they have cared about you and sorted things out. You get a confidence ... I was very sick with my third child with kidney problems. He used to say to me, 'Now don't worry, I'll be there' ... It was just that took me through the months, you know, knowing that he'd be there and looking after me sort of thing.' **Mrs M3 (diabetes)**

'She knows the kind of person I am, she knows that I don't moan about my health to her because I only go when it's something really that I can't deal with myself. ... If it's my own doctor, as I keep calling her, I immediately get into conversation with her, because well, I like her and that's that. But if it's a doctor I don't know or I've only seen maybe once before, I sit down at his desk and just wait for him to speak to me.' **Mrs T2 (high blood pressure)**

'When I go to [my doctor] he knows my case, he knows exactly what's what, where [other doctors] have to more or less look up everything. ... I think when you're seeing different doctors, I honestly feel they're only there to help you out as far as they can that day, because you're only seeing them that day.' **Mrs P2 (rheumatoid arthritis)**

Discussion of disadvantages of personal continuity was usually prompted, and focused on the risk of symptoms being taken for granted. Only two patients said they had experience of this actually happening, but both still said they saw the same GP. In that sense, it seemed to be a price worth paying for the perceived benefits. Rapid access was not said to have any disadvantages.

Negotiation of the appointment was largely done with the receptionist. Although this negotiation was discussed as sometimes problematic, particularly for patients in larger practices, only a few patients attributed these problems to the receptionists themselves usually because they resented discussing their 'problem' with a lay person. More commonly difficulties with access were attributed to general high demand, or 'their' GP being less available because part time or 'popular'. When negotiating appointments, most patients had one GP that they tried to see. If that GP wasn't available quickly enough, then most patients said they would see any other GP (although some had quite strong dis-preferences).

In summary, all patients were concerned with access because all had experience of having to negotiate it, predominately with receptionists. Under some important circumstances, the majority of patients balanced speed of access against the perceived benefits of consultation with a known and trusted GP.

### Personal continuity and access from the general practitioners' perpspective

GPs' discussion of their work focused on personal continuity as a central feature, including when discussing general practice in the abstract, when describing their own reasons for choosing it as a career, and in discussion of the care of individual patients. Like the patients, GPs said that personal continuity was particularly important when patients had multiple or complex problems, chronic disease, or psychological and emotional problems.

Personal continuity was said to allow more effective and efficient diagnosis and management of problems presented, because GPs considered them in the context of the whole person, including the patient's family and social circumstances, and their past response to illness. Less prominently, GPs said that seeing patients they knew was an important source of satisfaction with their work, and some said that patients liked to be seen by a doctor they knew and trusted (table 6).

**Table 6:** Why does personal continuity matter from the general practitioner's perspective?

'I think it's more important when people have say, a malignant condition or, a serious illness, a chronic illness.' **Dr U**

'I think it's most important for patients with ongoing illnesses for regular follow-up to be seen by the same person. The advantage of patients with ongoing illnesses seeing the same doctor is that hopefully you are already familiar with their pattern of illness, how it affects them, how they normally react to it, what a given symptom might mean to them.' **Dr M**

"I think most people staying with the same doctor, I think it helps. If you have a bit of background knowledge to people it will help you solve ... the problem a little bit quicker." **Dr C**

'Continuity of care is knowing the case and I think that's, it's particularly important in general practice because a lot of these things are not just to do with medical facts. But sometimes that's important that a patient will only see me, because when they see somebody else ... if things have gone off a bit you know, twenty five different drugs, where the hell do you start if you're seeing somebody else's patient like that, whereas your own, you probably can. So there's those sort of complicated patients, complicated in a technical medical sense. But also a lot of general practice is people explaining who they are, what they are, and what they're looking for out of the consultation. Particularly if they're kind of, either eccentric people or awkward people or whatever, then coming to a doctor who understands that is important.' **Dr P**

'And ultimately, I mean the job gets better because the longer you're in a practice, the sense of familiarity with it all and continuity, you know, it's nice seeing patients and their families coming in. ... It makes it more interesting when you can see the same problem happening, or you know where they've come from, you know how well they've done in life.' **Dr S**

Disadvantages were less commonly described, usually in response to a specific question. The main disadvantage identified was potentially missing slow change, such as a patient developing hypothyroidism. A few GPs said that too close a relationship risked doctors being unable to be objective about patient's problems, and could make patients less self-reliant and inappropriately dependent on the doctor.

Discussion of access was much less prominent than in the patient interviews, and unlike talk of personal continuity, was usually prompted by direct questions. Most GPs recognised that access could be problematic, and several of those with at least some open surgeries said they maintained these because they were popular with patients, although less so with doctors. For other surgeries, appointment making was largely delegated to receptionists, and GPs had little knowledge of how patients and receptionists negotiated this, although one GP suggested that receptionists and GP priorities might differ (table 7).

**Table 7:** Access discussed in response to specific questions, and not presented as central to GPs' work

'We have an ongoing problem with reception staff, because they see themselves very much as the patient's advocate and if a patient wishes an appointment on a Tuesday afternoon and I'm not available, they will feel that they're doing the right thing by the patient, by giving them an appointment with somebody else, so we have an ongoing problem in trying to get the receptionist to realise just how important it is to ask which doctor rather than what time.' **Dr T**

'I think appointment surgeries are an incredibly efficient way of seeing patients, and I also think that it's important that the patient can get seen on any day that they wish to. ... The disadvantage of an open surgery is that you get to see a whole crowd that probably would have got better anyway, the advantage is that patients really like it.' **Dr H**

'If they want to see one of the very heavily booked doctors they could wait two weeks to be seen. If they want to see, on average, it's maybe two days to see a doctor. The other factor to take into account is the time the patient wants to be seen. I actually did that when I was a student. I spent a week working out how long people had to wait for an appointment and the reasons why they had to wait and the single most important factor was the time didn't suit. The doctor might have lots of appointments, didn't suit, therefore (shrugs).' **Dr R**

In summary, GPs strongly emphasised the importance of personal continuity, and claimed benefits in terms of better diagnosis and management of chronic, complex and emotional problems. Discussion of access was usually prompted, and for most, the negotiation of access was not presented as a core part of their work, usually being delegated to receptionists.

### Do patients and general practitioners understand each others' perspectives?

There was little evidence that GPs and patients had discussed when personal continuity mattered, or how patients should balance the two when making appointments. Although several patients assumed that their GP valued personal continuity, only one patient had explicitly discussed this with their GP. Most patients said that they did not know what their GP thought. GPs generally assumed they knew what individual patients preferred, but this was not based on discussion with the patient. Rather it relied on observing patients' consultation pattern, which in some cases was misleading. Patient P3 predominately valued rapid access although she preferred the same GP on the few occasions when a particular problem required more than one consultation. However, in the last two years she had largely seen one GP because their work patterns had been congruent. The GP interpreted that as an indication of a strong preference for personal continuity. The reverse was also true. Patient M1 strongly preferred to see Dr M if possible, but could not usually do so because most of her consultations were for acute illnesses in her children where she prioritised access. Because she and her children saw a range of GPs, Dr M perceived that personal continuity was not important to her (table 8).

**Table 8:** Did patients and GPs know what each other thought about personal continuity and access?

*1. Lack of knowledge of GPs' beliefs by patients (both patients identified the interviewed GP as 'their' doctor, and both GPs said they thought personal continuity very important for chronic problems).* *'BG Is it something that Dr E has ever said to you – 'I think it's important that you see me?'* No it's just purely something that I am generating, the driver is mine, you know. It's entirely my desire to see her.' **Mr E3 (diabetes)**

*'BG You said quite strongly that you prefer to see the one doctor, do you think the doctors feel the same way, do they encourage you to stick to the same doctor?* Mmm now there's a thing. Eh, I never thought of that, eh, I suppose they do, would like you to stay with them. I'd hope so anyway.' **Mrs G3 (diabetes)**

*2. Misperceptions of patients' beliefs by GPs based on observing consultation patterns* 'I see Dr M if I can but it takes about 3 weeks to get an appointment ... so, like if she was ill, I'd want to see Dr M but it's impossible ... so usually I have to see one of the other doctors.' **Mrs M1 (no chronic disease)**

'As far as dealing with her on health problems in the past, I couldn't claim to know her history very well. She is the sort of person that if she came in I would have to have a good look through her records to have some understanding of what's been happening with her. ... *BG Is it a family where you think there is a main doctor, I mean is there a main doctor for her?* I don't think so, no, I wouldn't have thought so. I think they probably see one, maybe one or two out of the five of us, two maybe on a more regular basis and maybe one or two of the other doctors at other times.' **Dr M**

## Discussion

This study has used in-depth interview data to systematically explore the views of GPs and patients of the value of personal continuity and access to care. The study successfully recruited a heterogeneous sample of GPs, and of patients with experience of consulting with a range of problems and degrees of urgency. The proportion of patients saying they had a personal GP was comparable to large surveys, consistent with recruitment via the participating GP not preferentially selecting for patients that they knew very well (78% here vs 75%) [14]. One potential limitation is that the patients recruited had only a limited range of conditions. In particular, the study did not actively recruit patients with chronic diseases such as asthma where the need for urgent access is likely to be common, or patients with mental health problems where personal continuity may be particularly important. However, most of the patients in the study had had occasions where urgent access had been important to them, and several had consulted their GP with family problems, anxiety and depression. Patients with other conditions are also likely to balance when to be seen against who to see, although the choices they make in doing this will vary depending on the particular problems they are consulting with. A second potential limitation is that the study was limited to one UK region. However, sampling ensured a range of practice size and deprivation, and included practices from small towns and villages in Lothian as well as Edinburgh city. Survey research involving family practitioners in the UK, Holland and the US identifies personal continuity as an important feature of their clinical practice, although what patients value is less certain [5]. We therefore believe the results to be applicable elsewhere in the UK, although their generalisability to countries where healthcare organisation is radically different is less certain [6].

From the patients' perspective personal continuity and access to care were inextricably intertwined. In general, patients all valued rapid access. However, access to an appointment with a GP was a means to an end – help with the management of particular problems in a clinical consultation. Four of the patients interviewed considered that any GP could deal with their current and foreseeable problems, and only valued rapid access to an appointment. The rest identified previous, current or foreseeable problems where seeing a known and trusted GP familiar with their past history was important. What patients therefore wanted was 'access to appropriate care' [24], where what was appropriate depended on the problem to be dealt with. For chronic, complex and psychological problems this was usually consultation with a GP with whom the patient had an ongoing relationship, because it made the process of consultation easier, and allowed greater involvement in decision making. For minor or episodic problems, or where the problem was perceived as very urgent, then any GP was likely to be appropriate.

The GPs agreed that personal continuity mattered under broadly the same circumstances, although they focused on its value in improving the diagnosis and management of problems in the face-to-face consultation. In the GP interviews, there was little unprompted discussion of access to care or the negotiation of appointments, which was therefore presented as much less central to their work. A key finding is that patients and GPs appeared to have little explicit knowledge about the other's perceptions of personal continuity and access. GPs' beliefs about what patients valued appeared based on their consultation pattern, which could be misleading since it was contingent on both patient preferences and other circumstances such as the problems they had had, and the way that appointment systems were organised.

General practice research and training have placed great emphasis on the consultation, as the place where the core values of personal continuity and holistic care are played out. The GPs interviewed had internalised this, but the emphasis on appropriate care *in *the consultation was at least partly at the expense of paying inadequate attention to access *to *that consultation.

Equally though, current policy focuses almost exclusively on rapid access *to *consultation without considering or measuring effects on the appropriateness of care *in *the consultation. Although Advanced Access documentation emphasises that patients should be able to book appointments with the clinician of their choice, the measures used to judge successful implementation, and the targets set for primary care organisations focus almost exclusively on 'entry access' or the speed with which patients get into the system [11,10]. There is some evidence that pressure to achieve these targets reduces personal continuity, and therefore leads to less appropriate care in the consultation for those with more complex problems [24,10,12], although the extent and implications of this will remain uncertain until external evaluation of Advanced Access is complete [25].

The evidence presented here supports changes to everyday practice and current policy in relation to access arrangements to primary care. However, the patients' perspective would be better accommodated if *both *GPs and policy targets addressed 'access to appropriate care' (or in-system access), rather than focusing on personal continuity (GPs) or rapid access (policy) [24]. All patients in this study wished rapid access under some circumstances. However, for ongoing and complex problems with less immediate urgency, then access to appropriate care for most patients meant being able to see 'their' GP who knew them as an individual, and knew their medical history. From this perspective, demand management strategies focused on improving speed of access including triage and direction of patients to the first available provider increases professional control over the definition of what care is 'appropriate' and reduces patients' ability to choose the service that they consider appropriate for the problem they wish to discuss.

## Conclusion

Ensuring access to appropriate care (including the facilitation of personal continuity to those who want it) will require general and family practice to continue the process of making access a core value [24,26], and for GPs to make access part of their everyday work. If GPs truly value personal continuity, then they need to ensure that they and their practice organisation facilitate it on a day to day basis. Strikingly, although many GPs and patients appeared to 'agree' about the importance of personal continuity for that patient, only one patient had explicitly discussed this issue with their doctor, and this discussion had been prompted by the patient. Generally GPs relied on observing patients' pattern of consultation to infer whether personal continuity mattered to that patient. In two of the interviews here, clearly incorrect inferences were made. Promoting personal continuity for those who want or need it would be better achieved by GPs discussing with patients whether and when they believe that patient should seek personal continuity, and ensuring that their system of access allows patients to then exercise realistic choices between 'who to see' and 'when to be seen'.

Equally, appropriate service re-organisation would be facilitated by policy targets that are based on more than simplistic measures of entry access. This will require the development of more sophisticated measures that capture the appropriateness of the care delivered as well as the speed of access to it, and the incorporation of these measures into targets and performance management systems. This study indicates that existing provider continuity measures based on averaging patterns of consultation over time do not capture the way that patients balance access and personal continuity for each consultation depending on the problem to be addressed. Measures of access to appropriate care are therefore likely to require that patients are asked to describe and rate both speed and convenience of the appointment, and whether they are consulting with their preferred clinician.

## Competing interests

The author(s) declare that they have no competing interests.

## Authors' contributions

BG conceived the study, participated in its design, collected the data, was the primary analyst and wrote the first draft of the paper. SW participated in the design of the study, contributed to analysis as part of the project team and analysed a selection of transcripts, and commented on and revised initial drafts of the paper.

## Pre-publication history

The pre-publication history for this paper can be accessed here:



## Supplementary Material

Additional File 1 Interview topic guides. Examples of topic guides used at the start of the main phase of the study (the main later modification was the addition of further probes about 'trust' and 'confidence' if participants did not spontaneously mention them) Click here for file
