# Supplementary material for: Personal continuity and access in UK general practice: a qualitative study of general practitioners' and patients' perceptions of when and how they matter
Source: BMC Fam Pract. 2006 Feb 24;7:11. doi: 10.1186/1471-2296-7-11 (PMC1413534; doi:10.1186/1471-2296-7-11)
Supplement: Additional File 1 — Interview topic guides. Examples of topic guides used at the start of the main phase of the study (the main later modification was the addition of further probes about 'trust' and 'confidence' if participants did not spontaneously mention them) [file 1471-2296-7-11-S1.doc]

# Additional file 1 - Interview topic guides

# GP interview topic guide

Date

Doctor ID

Sex

Age

Length of time in this practice

Actual number of sessions worked in practice

Other practice experience

List size and ranking

Deprivation score and ranking

Partners’ names according to medical list. Ask for sex, full/part time and years here

1.

2.

3.

4.

5.

6.

7.

8.

9.

10.

11.

12.

How many nurses including full and part time

Training practice

Appointment system or open surgery arrangements

Out of hours arrangements

**Introduction**

I’d just like to reiterate that everything you say in the interview is confidential to me and my supervisors. If anything you say is used in a publication then it will be anonymous. The interview itself will be open ended and the questions themselves are usually fairly broad. There aren’t any right or wrong answers - I’m simply interested in your experience and your views. The first part of the interview is about your experience of being a GP in general and then later on I’ll be asking you about particular patients and their care. Is there anything you’d like to ask me?

**Biography**

Can you tell me how you came to be a GP?

Can you tell me how you chose this practice?

**Defining general practice**

What do you think makes general practice what it is?

Why are these areas important? (doctor identified)

What do you think are the most important aspects of what you’ve identified?

Why have you chosen these ones?

How do you think you have developed your views of what makes general practice what it is?

How have things changed in the time that you’ve been working in this practice?

How has your personal experience of work changed?

How have your relationships with patients changed?

How have your professional relationships changed?

What’s changed for the better?

What’s changed for the worse?

How do changes in one area affect another?

What do you see as changing in the next few years?

**Continuity**

In the general practice context, what do you think is meant by the term “continuity” or “continuity of care”? How would you define continuity in general practice? You mentioned “continuity”, can I just check what you mean by that?

Is continuity important?

In the wider scheme of things, what is the place of continuity?

How does continuity relate to these others?

How important is it in relation to the others?

Is it/its elements important to everyone?

**Contrast with other settings and professionals**

How do you think being a GP differs from being a hospital doctor?

The role of nurses in primary care has changed and looks likely to keep changing? How do you think this affects your work?

How does the work of receptionists impact on what you do?

**Personal experience**

How well do you think you provide ‘continuity of care’? (?several questions)

When defining continuity earlier, you talked about XXX, but you haven’t (really) mentioned it here...

Compared to other doctors, how well do you think you provide it?

Can you give some examples of it in your personal practice?

Can you give some examples of when it’s broken down?

What are the mechanisms by which you encourage or discourage it?

What difference does continuity make to your own clinical practice or experience of work?

How well do you think the practice provides ‘continuity of care’?

Can you give some examples of it in action in this context?

Can you give some examples of when it’s broken down?

What are the mechanisms by which the practice encourages or discourages it?

What difference does it make to the way the practice works?

If we could now move on to the individual cases.

**Patient 1**

Can we start with……? Could you tell me about them?

How would you describe your relationship with this person?

Do you think that’s fairly typical for you?

How would you describe this person’s relationship with the practice?

Do you think that’s fairly typical?

Which aspects of their care do you think have been good?

Are there any aspects of their care which you think have been less satisfactory?

When I asked you to find names, I asked that the diabetics/hypertensives be someone that you knew well/you have some personal experience of but whom you wouldn’t say that you know well. Can I ask what you mean by ‘knowing well’/’not knowing well’?

I’d like to relate what we were talking about earlier in general to this particular patient. We talked about XXXXX and XXXXX.

How do you think those general ideas are reflected in this person’s care?

Is there anything you’d like to add to what we’ve discussed?

**Patient 2 etc**

Shall we move onto …….? Could you tell me about them?

# Patient interview topic guide

Date

Identifier

Can I just make sure that I’ve got some details about you correct?

Can I just check how old you are?

Sex

What would you say your main health problems are?

How long have you been registered with this practice?

How often you go to see your GP?

In the last year say, how often have you been?

Who lives with you?

Are you working at the moment?

What do you do?

Is your partner working at the moment?

What does he/she do?

Do you have family living nearby?

Who?

Where?

Thank you for agreeing to help with this research. The aim of it is to find out what patients and GPs think are the most important aspects of general practice care to try and improve what happens in the future. Everything that you say will be confidential. Your own doctor won’t find out anything you say. It’s possible that some quotes from this interview might be used in publications, but if that happens then they will be anonymous - they won’t identify you or the practice by name. Is there anything you want to ask me about?

The style of the interview will be open ended so you may find some of the questions quite broad or quite difficult. There aren’t any right or wrong answers. I’m interested in what you think and feel.

**Choosing the practice**

How long registered here? How did they choose it? Did you consider any other practices?

**Using the GP**

Is there anything they go to the GP regularly about? If not now, then what about in the past?

Any particular health problems including things they don’t go to the GP about?

**Particular experiences**

I’d like to talk a bit more about your own experience of seeing doctors and ask you some questions about particular times that you’ve gone to see your doctor. I’d like you to think about the last time you went to see your GP (or any recent contact if most recent is very focused eg getting a passport application signed).

What made them decide to go then?

What happened when rang to get appointment? Do the receptionists know you and does this help?

Do they describe themselves as being active?

What happened afterwards?

Which doctor did they see? Why this doctor? (Choice, availability, own doctor not available, special expertise)

**General questions about GPs**

I’d now like to ask you some questions about doctors and general practice. These questions are about things in general rather than about your actual doctors. You may find these questions a bit vague or a bit difficult.

I’m interested in knowing from your own experience what would make a doctor seem good or not so good or bad for you. So just in general, could you describe what you think a good doctor is like or what the ideal doctor for you would be?

Do you think it always works like that? Could you be good and not be like that? Have you ever come across doctors like that?

The next question is asking you to look at it the other way. Can you describe what you think a bad doctor is like or what the worst doctor for you would be?

Have you ever come across doctors like that?

Overall, what do you like about general practice? What’s it done for you that’s been good?

Is there anything about it which you would change if you could? When does it annoy you, or make it difficult for you to get what you want or need?

**Experience of other settings**

Have they any experience of other practices? How were they different?

Have they any experience of hospitals? How are they different?

Have they seen nurses at all? How is that different from doctors?

**Choosing doctors**

I think you said that you’d seen Dr. X, so…

When they go to the doctor, do they have a particular doctor or doctor (or doctors) that they try to see? Why? How does this relate to access? What is it about them that makes them want to see them? Can they give examples of when it’s been important to see a particular doctor? And not important? If never important, then what is most important when organising the appointment?

Are there any particular GPs that they prefer not to see? Why? What is it about them that makes them not want to see them? Do you ever end up seeing doctors that they’d prefer not to see? Why? Can they give an example of when that’s happened?

Does the practice try to make them stick to the same doctor? What do they think about that? Do the doctors ever suggest that they come back to them in particular?

**My friend John questions**

Some people say that they like to see the same doctor most of the time, others don’t mind and others like to see different doctors. What do you think about that?

Some people (doctors/patients) say that it doesn’t matter which doctor you see, because they’ve all got the notes/a written record. What do you think about that?

Some people who don’t mind which doctor they see say that this means that they can see a doctor more quickly than waiting for a particular one. What do you think about that? Do you ever wait for a particular doctor or do you usually just want to be seen quickly?

Some people say that what matters is that they see a doctor who knows them. Is that important to you? What do you mean by knowing them? What sort of knowledge?

GPs often say that they think it’s important to look after families. Does your GP know your family at all? Do you think that’s important for you? Or the family? Or the doctor?

**What kind of person?**

Some people say that as well as doctors knowing their medical details, it’s important that doctors know what sort of person they are as well. Is that something that’s important for you?

Does your doctor know what sort of person you are?

Do you think that all the doctors in the practice know that or just some?

How do you let them know that? How do they find things like that out?

**Atrocity and angel stories**

Are there any visits to your GP that stand out in your mind, perhaps because you were very ill or very worried, or perhaps because things went well or not so well?

Why do you think that was?

What about things you’ve heard other people talk about, perhaps family or friends describing what’s happened to them?

Went well vs went badly

**Specific health problems**

Could you tell me more about....? (based on health problems they identify)

What happened when it was diagnosed? Do they wish that anything had been done differently? What’s happened since then? What would you like to happen in the future?
